# Controlling malaria in Niger with bednets: how to take the Big Picture

**DOI:** 10.1186/1475-2875-9-S2-O12

**Published:** 2010-10-20

**Authors:** Duchemin Jean-Bernard, Czeher Cyrille, Labbo Rabiou, Ibrahim M Lamine, Hoyer Stefan, Jeanne Isabelle

**Affiliations:** 1 CERMES, BP 10887, Niamey, Niger; 2 Genetics and Genomics Insect Vectors Unit, Institut Pasteur, 25-28 rue du Dr Roux, 75724 Paris Cedex 15, France; 3 Global Malaria Programme, World Health Organization, 20 Avenue Appia, CH 1211 Geneva 27, Switzerland

## Background

In December 2005 free long lasting insecticidal nets were distributed to mothers of children under five in a nation-wide scheme in Niger. More than 2 million bed nets were distributed, increasing the ownership of insecticide treated bed nets more than tenfold.

## Materials and methods

Our team was in charge of the malaria survey. The malaria cases were reported through a network of 44 sites across the country. Malaria transmission was surveyed in 12 villages in the Sahel, as it was deemed the most variable zone.

## Results and discussion

During the first year of follow-up, vector dynamics showed an overall decrease in malaria transmission, but this was highly variable from village to village. Indeed, the second year of survey showed a return to transmission levels close to the pre-intervention period. A study of microsatellite markers showed no modification of the genetic structure in the two malaria vectors *An. gambiae* and *An. arabiensis.* However, a clear increase of the resistant allele of the kdr gene - resistence to the pyrethroid insecticide used in the bed nets - was observed. This clearly demonstrated that the main target vectors were reached. As measured by ovarian tracheoles observation, the parity rate of vectors was not significantly decreased. However, the index sporozoite decreased in the first year and remained low in the second year. Indeed, the parasite prevalence and the gametocyte carriage were shown decreasing in the children under five. This continued in 2007 as the National Malaria Control Program provided free artemisinin-based combination therapies as first line treatment for malaria in children under five. The national survey network showed a decrease in malaria cases, as confirmed in our sentinelle sites. However the biological confirmation of cases by HRP2 plasmodial antigen research has increased during the two years of follow-up. This could be explained by the more accurate diagnosis by peripheral health personal with the help of rapid diagnostic tests.

## Conclusion

Our survey system allowed the report of the malaria evolution during that important period of control reinforcement in Niger. The causative impact was difficult to confirm because several lines of malaria control have been successively implemented. Moreover, the high climatic variability in this zone needs to be taken in account in the analysis. Despite these difficulties our data reinforce the importance of malaria monitoring through multidisciplinary studies to the benefit of control operations.

